# Proteomic study of the membrane components of signalling cascades of *Botrytis cinerea* controlled by phosphorylation

**DOI:** 10.1038/s41598-019-46270-0

**Published:** 2019-07-08

**Authors:** Almudena Escobar-Niño, Eva Liñeiro, Francisco Amil, Rafael Carrasco, Cristina Chiva, Carlos Fuentes, Barbara Blanco-Ulate, Jesús M. Cantoral Fernández, Eduard Sabidó, Francisco Javier Fernández-Acero

**Affiliations:** 1 Andalusian Center for Grape and Grapevine Research (IVAGRO), Microbiology Lab, University of Cadiz, Puerto Real, 11510 Spain; 2 Bioinformatics Unit, SCAI, University of Córdoba, Ramón y Cajal Building, Rabanales Campus, 14071 Córdoba, Spain; 3 Proteomics Unit, Centre for Genomic Regulation (CRG), 08003 Barcelona, Spain; 4 Proteomics Unit, Universitat Pompeu Fabra (UPF), Barcelona, 08003 Spain; 5 Proteomics Unit, SCAI, University of Córdoba, Ramón y Cajal Building, Rabanales Campus, 14071 Córdoba, Spain; 6 Department of Plant Sciences, University of California, Davis, CA 95616 USA

**Keywords:** Protein-protein interaction networks, Fungal pathogenesis

## Abstract

Protein phosphorylation and membrane proteins play an important role in the infection of plants by phytopathogenic fungi, given their involvement in signal transduction cascades. *Botrytis cinerea* is a well-studied necrotrophic fungus taken as a model organism in fungal plant pathology, given its broad host range and adverse economic impact. To elucidate relevant events during infection, several proteomics analyses have been performed in *B*. *cinerea*, but they cover only 10% of the total proteins predicted in the genome database of this fungus. To increase coverage, we analysed by LC-MS/MS the first-reported overlapped proteome in phytopathogenic fungi, the “phosphomembranome” of *B*. *cinerea*, combining the two most important signal transduction subproteomes. Of the 1112 membrane-associated phosphoproteins identified, 64 and 243 were classified as exclusively identified or overexpressed under glucose and deproteinized tomato cell wall conditions, respectively. Seven proteins were found under both conditions, but these presented a specific phosphorylation pattern, so they were considered as exclusively identified or overexpressed proteins. From bioinformatics analysis, those differences in the membrane-associated phosphoproteins composition were associated with various processes, including pyruvate metabolism, unfolded protein response, oxidative stress response, autophagy and cell death. Our results suggest these proteins play a significant role in the *B*. *cinerea* pathogenic cycle.

## Introduction

*Botrytis cinerea* is one of the most destructive phytopathogenic fungi^1,2^ since it can infect a wide range of commercial crops. It has a dual role as both a parasitic and a necrotrophic fungus, and given its life cycle it has been taken as a model in fungal plant pathology. The great versatility of *B*. *cinerea* derives from its ability to adapt its infective cycle to the perceived environmental conditions, using a diverse set of virulence/pathogenicity factors to attack plant tissues and suppress plant defence^2,3^.

In the post-genomic era, proteomics studies have become a crucial tool to understand the molecular mechanism involved in a wide range of biological processes. The total number of articles on fungal plant pathogen proteomics is still increasing slowly^4–7^. Several studies have highlighted the changes that take place in the *B*. *cinerea* proteins during its infection cycle, as well as their relationship with known pathogenicity factors, and the role of “moonlight proteins”, e.g. GADPH, in the fungal proteome^8^. However, the proteins identified in proteomics studies of *B*. *cinerea* only cover around 10% of the total proteins predicted in the genome database^9^. In an attempt to increase this coverage, several different subproteomes have been characterized, particularly phosphoproteins^10,11^, and membrane proteins^12^. To further investigate the protein components of transduction pathways, a study has been undertaken to obtain the subproteome (phosphoproteins) of a subproteome (membrane proteins) with the object of revealing those membrane proteins that are controlled by phosphorylation. We have named this subproteome the “phosphomembranome” of *B*. *cinerea*. As far as we know, this is the first overlapping proteome approach reported in the study of phytopathogenic fungi.

In *B*. *cinerea* the infection process requires efficient communication between its biochemical machinery and the surrounding environment. This dialogue is mediated by signal transduction cascades, such as the Small GTPase, Ca/calmodulin-dependent and MAPK signalling pathways, which transduce the perceived external conditions into intracellular signals. In most cases these involve phosphorylation events^13^. It has been established that, among the known post-translational modification (PTM) processes, phosphorylation is the main mechanism of signal transduction and modification of enzymatic activity in living organisms^14^. Furthermore, this PTM has been reported to have an important role in infection processes of fungal plant pathogens, including *B*. *cinerea*^13,15^. It is also known that membrane proteins play an important role as receptors and transporters activating signalling cascades that connect the fungal response to changes in the environment^16,17^. In *B*. *cinerea*, most of the pathogenicity/virulence factors that have been described are related to phosphorylation/dephosphorylation activity, (http://www.phi-base.org)^18^; these include MAP kinases, the RAS superfamily, GTPases and histidine kinases^19–21^. About 30% of all virulence factors are integral membrane proteins with transmembrane alpha-helix (TMH) domains^22,23^.

All this existing knowledge points to the importance of both the membranome and the phosphoproteome of *B*. *cinerea* for understanding key points in the regulation of the infection process. In this paper, for the first time, we present a combined analysis of both subproteomes (now jointly named the “phosphomembranome”), with the objectives of increasing the coverage of membrane proteins identified, and of elucidating the main components of the transduction pathways that control the infection process. Towards these goals, we have analysed the phosphorylation of membranome proteins in *B*. *cinerea* under different virulence conditions, induced by plant-based elicitors, including glucose (GLU) as a constitutive stage, and deproteinized tomato cell wall (TCW) as a virulence inductor. Both conditions are known to induce different fungal responses in the *B*. *cinerea* phosphoproteome and membranome^11,12^. Interestingly, the differences identified in the composition of membrane-associated phosphoproteins in each assayed condition may indicate the relative importance of the regulation of particular factors - the pyruvate metabolism, unfolded protein response (UPR), oxidative stress response, autophagy and cell death - during the infection process of *B*. *cinerea*.

## Results and Discussion

### Membrane phosphoprotein identification

Differences in the phosphomembranome of *B*. *cinerea* were analysed using mycelium harvested under two different pathogenicity stages: glucose (GLU) as a constitutive stage, and deproteinized tomato cell wall (TCW) as a virulence inductor, as has been previously described^11,12^. Membrane proteins were isolated and phosphorylation was enriched as described in the Methods section. After LC-MS/MS analysis, a total of 2318 phosphopeptides (2073 different peptides) were identified under the two conditions assayed (Supplementary Data Table S1), belonging to a total of 1112 membrane-associated phosphoproteins. In order to provide greater consistency to our comparative study only those proteins identified in at least three of the four biological samples in each assayed condition were considered for further analysis. According to this, a total of 580 phosphopeptides were considered in our research. After qualitative analysis, 64 phosphoproteins (72 different phosphopeptides, 74 including specific phosphorylation) were exclusively identified or overexpressed under GLU conditions (Supplementary Data Table S2a) and 243 phosphoproteins (324 different phosphopeptides, 327 including specific phosphorylation) exclusively identified or overexpressed under TCW conditions (Supplementary Data Table S2b). Seven phosphoproteins found under both GLU and TCW conditions, exhibited specific phosphorylation patterns for each condition, and therefore, they were also considered as phosphoproteins exclusively identified or overexpressed (Supplementary Data Table S2). The mass spectrometry data obtained show differences in the composition of the phosphomembranome in *B*. *cinerea* depending on the carbon source assayed. The mass spectrometry proteomics data have been deposited in the ProteomeXchange Consortium^24^ via the PRIDE partner repository, with the dataset identifier PXD010961.

**Figure 1:**
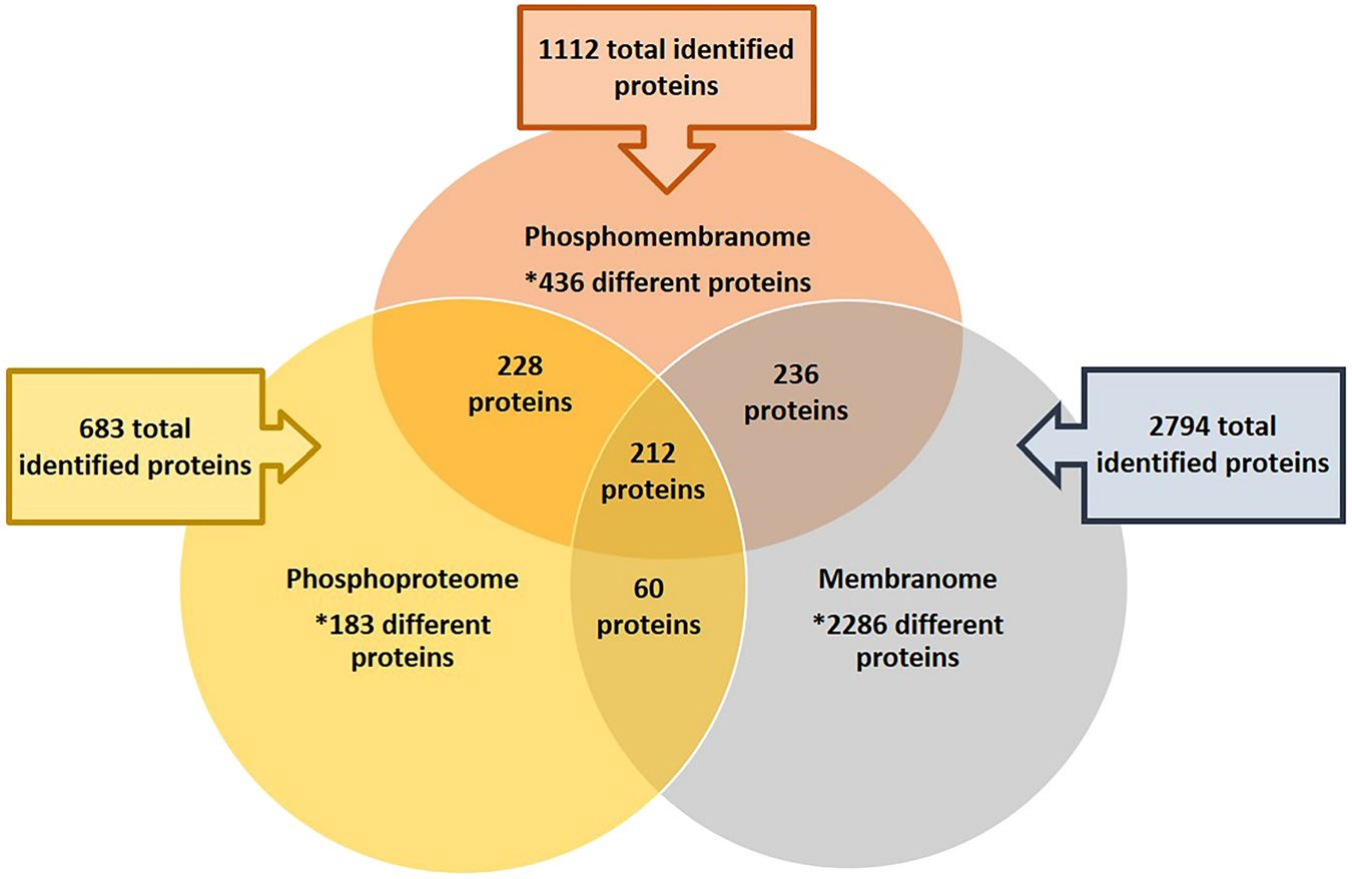
Comparative analysis. Comparative analysis between the total identified proteins in Phosphomembranome (pink), Phosphoproteome (orange) and membranome (blue) of *Botrytis cinerea*. The overlaps between circles represent the common identified proteins among these subproteomes.

Comparing the 1112 membrane-associated phosphoproteins identified in this work with those proteins identified in our previous proteomics analysis, the *B*. *cinerea* phosphoproteome and membranome^11,12^, a total of 436 (39.21%) new proteins have been identified (Fig. 1). These new proteins mean a 2.6% increase in the 10% of the total proteins predicted in the genome database^9^ covered by the previous proteomic analyses. Focusing our attention only on those proteins exclusively identified or overexpressed under each assayed condition, the comparative analysis showed 7 (10.94%) and 123 (50.62%) proteins identified under GLU and TCW, respectively, which were not identified in the phosphoproteome or the membranome (Supplementary Data Table S3). Between the proteins exclusively identified or overexpressed under each condition only 7 were among the pathogenicity/virulence factors described at (http://www.phi-base.org): (i) 2 of the proteins exclusively identified or overexpressed under the GLU condition (Bcatg3 and BcSAK1); (ii) 5 of the proteins exclusively identified or overexpressed under the TCW condition (BcCLA4, BcSAK1, BcCRZ1, Bos5 and Bcbrn1). Putting all these results together, it is evident that the phosphomembranome has been a useful approach to identify new proteins potentially implicated in the pathogeny/virulence of *Botrytis cinerea*.

### Topology analysis of membrane-associated phosphoproteins

The phosphoproteins identified were classified based on topology prediction algorithms to determine the protein’s role as a membrane component. Integral membrane proteins were identified by predicting the presence of transmembrane α-helix domains (TMHs). Proteins without predicted trans-membrane domains were further analysed to find other relationships with the membrane, such as the presence of secretory signal peptides or other covalent associations (palmitoylation, prenylation, myristoylation and GPI anchor). Proteins predicted to include these modifications were considered to be potentially associated membrane proteins, as has been previously described^12^. The proteins not assigned to any of the previously-mentioned categories were classified as peripheral and/or cytosolic proteins.

**Figure 2:**
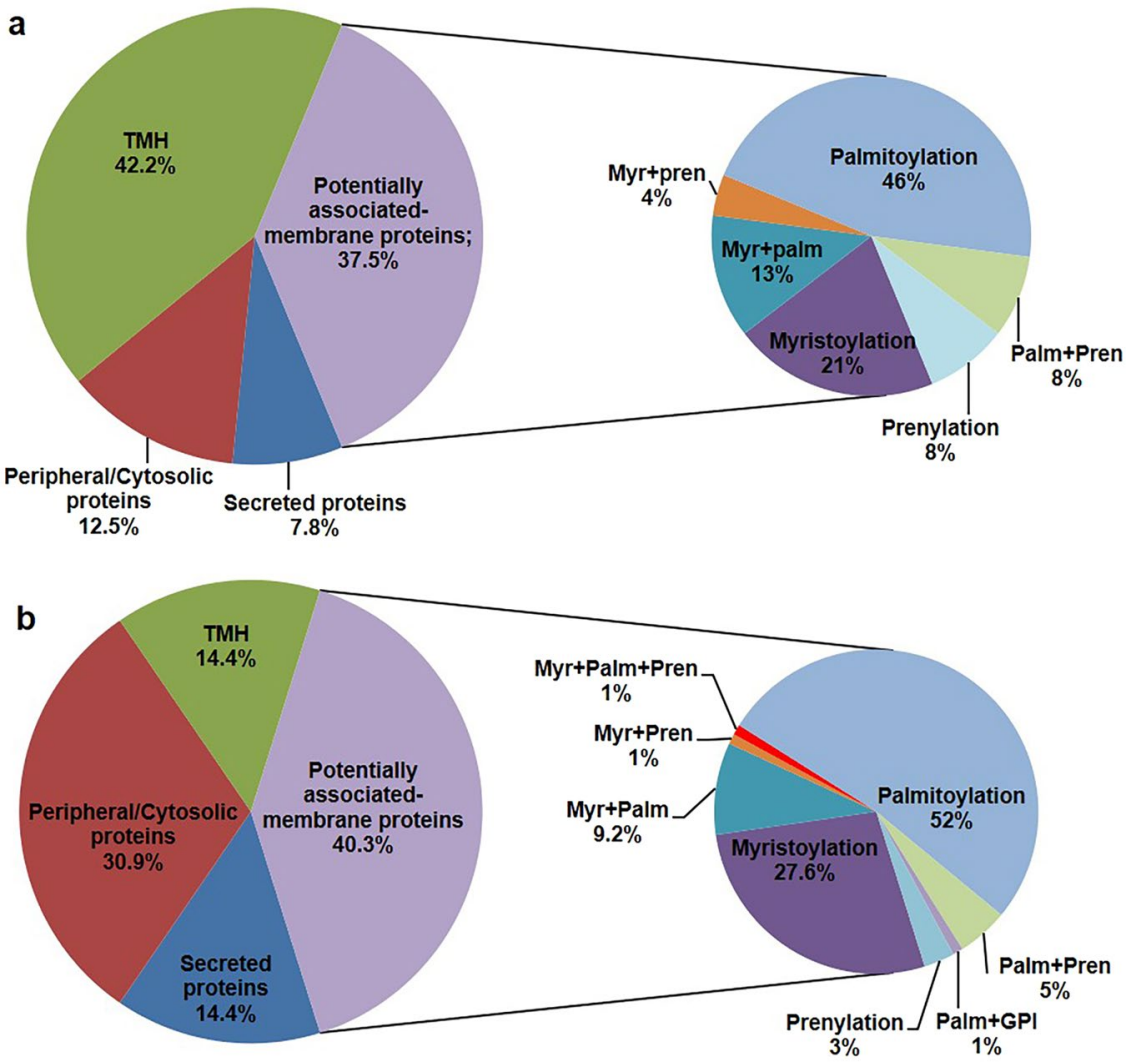
Topological classification. (**a**) Exclusive and over-expressed proteins under the GLU condition; and (**b**) Exclusive and over-expressed proteins under the TCW condition. Keys: TMH = integral membrane proteins; Myr + Pren = myristoylation and prenylation sites; Myr + Palm = myristoylation and palmitoylation sites; Palm + Pren = palmitoylation and prenylation sites; Myr + Palm + Pren = palmitoylation, myristoylation and prenylation sites; Palm + GPI = GPI and palmitoylation sites; Myristoylation = myristoylation sites; Prenylation = prenylation sites; Palmitoylation = palmitoylation sites.

Based only on the presence of predicted proteins with TMHs, the topological prediction analysis showed that integral membrane proteins with TMHs account for 42.2% and 14.4% of the proteins exclusively identified or overexpressed under GLU and TCW, respectively (Fig. 2a,b). Because this kind of approach is new in fungal proteomics, the comparison of our data with other fungal species is not possible. A previous reported analysis using human microsomes from K562 cells showed that only around 30% of the membrane proteins isolated have transmembrane regions; this could imply that a large number of non-integral proteins are associated in some way with membranes, suggesting a significant role for peripheral proteins and lipidated proteins in the biology of organisms^25^. Further, of exclusive or overexpressed proteins, 7.8% and 14.4% were classified as secreted proteins under each condition, GLU and TCW, respectively; 37.5% and 40.3% of exclusive or overexpressed proteins were classified as proteins potentially associated with the membrane by lipidation, under each condition, GLU and TCW, respectively; and the remaining exclusive or overexpressed proteins, 12.5% and 30.9%, were classified as peripheral/cytosolic proteins under GLU and TCW, respectively (Fig. 2a,b). Of the predicted proteins potentially associated with the membrane under each condition, the analysis showed that palmitoylation is the most typical lipidation in both conditions (Fig. 2a,b); this finding agrees with our previous analysis of the membranome of *B*. *cinerea*^12^. Taking all the identified proteins with positive signals for lipidation together with secreted proteins (since many secreted proteins can be anchored to the membrane during their secretion^26^), the percentage of identified membrane-associated proteins increases from 42.2% to 87.5% under the GLU, and from 14.6% to 69.1% under the TCW condition. Comparing this topological prediction to our previous results in the analysis of the membranome of *B*. *cinerea*, the percentage of predicted membrane proteins is higher, especially under GLU conditions, at 87.5% compared with 42% under GLU, and at 69.1% compared with 63% under TCW^12^.

For each condition assayed, the transmembrane proteins exclusively identified or overexpressed under each condition were classified by their number of predicted TMH domains. These numbers were then compared with the predicted profile of TMH-containing proteins present in the *B*. *cinerea* genome database^27^, and with our previous results regarding the membrane proteome^12^. The number of proteins with TMH domains in the phosphomembranome of *B*. *cinerea*, identified under our conditions, cover only 3.7% of the total of 1659 proteins with predicted TMH domains in *B*. *cinerea*, compared with 28% of the proteins with TMHs identified in our previous analysis of the *B*. *cinerea* membranome^12^. In spite of the low coverage, these results show a similar distribution in terms of number of TMHs per protein, compared with proteins present in the *B*. *cinerea* genome database, which span the range from 1 to 14 TMHs (Supplementary Information Fig. S1); this finding shows that our membrane protein isolation and phosphopeptide enrichment protocols do not produce any artificial increase in the number of transmembrane domains. This result also shows that there is no special relationship between the number of TMHs and protein phosphorylation.

### Analysis of phosphorylation site variation depending on the carbon source

**Figure 3:**
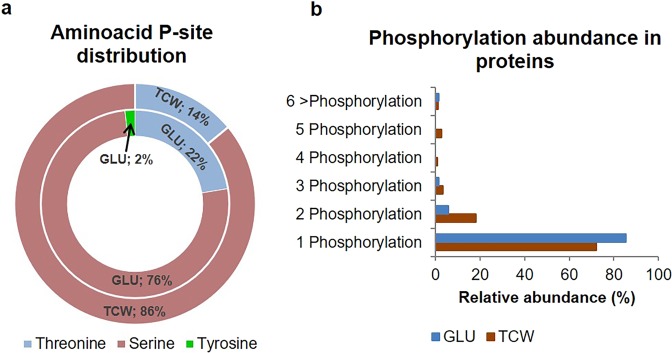
Distribution of phosphorylation sites identified. (**a**) Distribution of phosphoproteins presenting a unique P-site by the phosphorylated amino acid: (i) external circumference represents the percentage of phosphoproteins with a unique P-site in Serine (red), Threonine (blue) or Tyrosine (green) under the TCW condition; (i) internal circumference represents the percentage of phosphoproteins with a unique P-site in Serine (red), Threonine (blue) or Tyrosine (green) under the GLU condition. (**b**) Frequency distribution of phosphoproteins according to the number of phosphorylation sites identified.

The differences in the number of phosphopeptides and in the phosphorylated site (P-Site) pattern between the assayed conditions was studied. Under the GLU condition, 58 of the 64 exclusive or overexpressed phosphoproteins identified presented a unique P-site, of which 44 (76%) were found in serine, 13 (22%) in threonine and 1 (2%) in tyrosine residues. Similar results were obtained under the TCW condition, in which 178 of the 243 phosphoproteins presented a unique P-site, with 153 (86%) phosphorylated in serine and 25 (21.42%) in threonine (Fig. 3a). More than 70% of the identified phosphorylated membrane proteins contain only one P-site (Fig. 3b), and the largest number of unique phosphorylation P-sites are in serine; these results agree with our previous data on the *B*. *cinerea* phosphoproteome^11^. Our results report differences in P-site distribution depending on the available carbon source in our study conditions; the largest number of serine phosphorylated proteins were found under TCW; Proportionately more threonine phosphorylated proteins were found under GLU than under TCW; and no unique tyrosine P-sites were found under TCW conditions. Compared to previously reported result for *B*. *cinerea*, *A*. *brassicicola*^10^ and *C*. *albicans*^28^, grown in culture media with mixed carbon sources, our data showed slightly higher phosphothreonine levels under GLU conditions and lower phosphotyrosine levels under TCW conditions. This confirms that phosphorylation of signal cascades components plays an important role in the regulation of *B*. *cinerea* metabolism with the object of adapting to its particular environmental conditions, such as a different carbon source.

Among the exclusive or overexpressed phosphoproteins identified, 7 proteins were common to both assayed conditions but presented different patterns in phosphorylation, with different phosphopeptides and/or different phosphorylation sites found in the same phosphopeptide depending on the assayed carbon source (Supplementary Data Table S2). These variations in the pattern of phosphorylation could affect the activity of proteins, and suggests specific regulation of the biological processes in which these proteins are involved via post-translational modifications. Four of these proteins were identified as: (i) stress-activated mitogen-activated protein kinase SAK1; (ii) acetyl-CoA carboxylase; (iii) putative major protein of the Woronin body; and (iv) pyruvate carboxylase. It is notable that two proteins, acetyl-CoA carboxylase and pyruvate carboxylase, involved in pyruvate metabolism are present. This finding, taken with the results of the subsequent analysis of clusters (MCODE), the GO and KEGG annotation, could indicate that pyruvate metabolism regulation plays an important role in the adaptation of *B*. *cinerea* to changes in environmental conditions, and therefore in the overall pathogenesis process.

### Gene ontology classification

**Figure 4:**
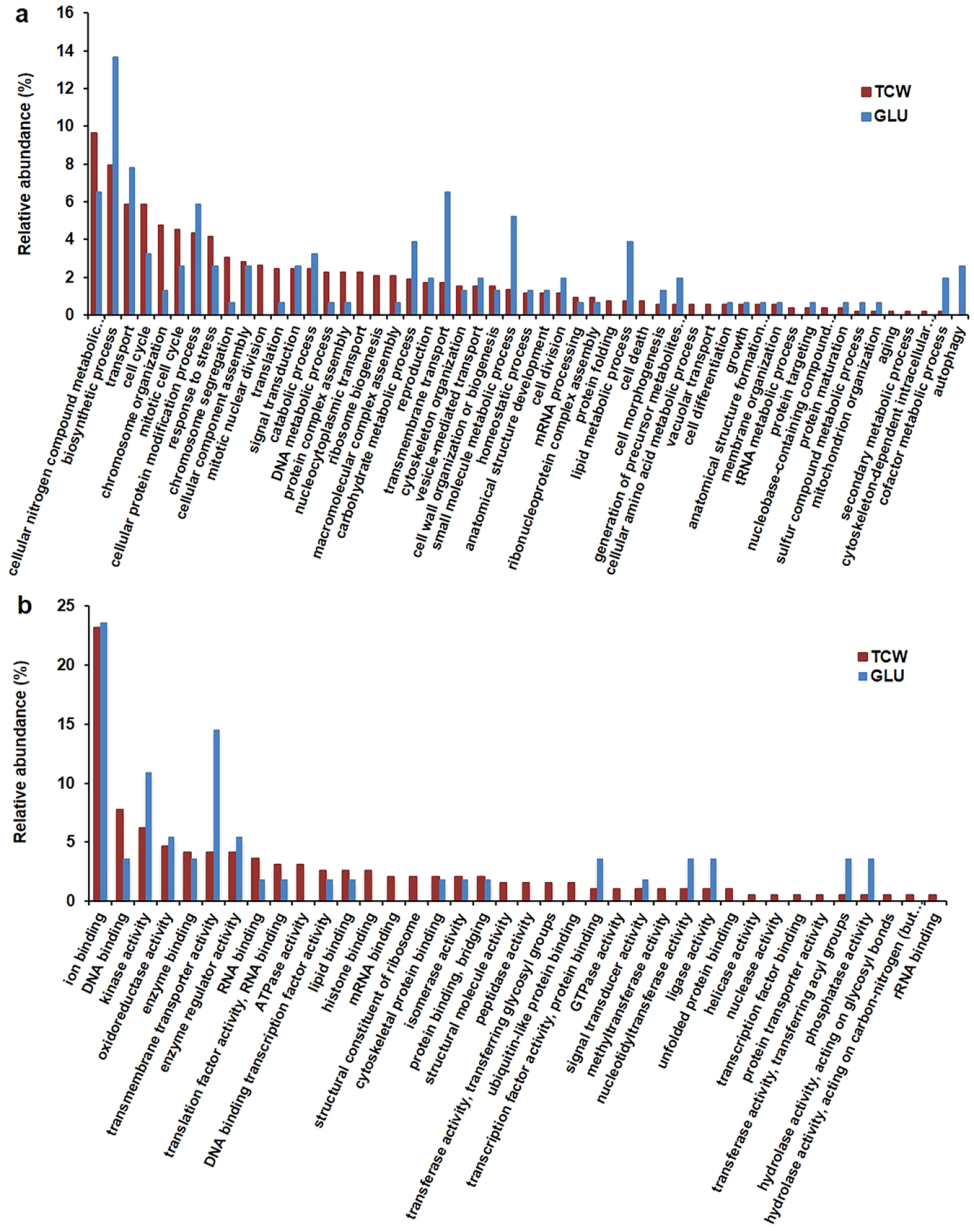
Gene ontology (GO) classification of membrane-associated phosphoproteins exclusively identified or over-expressed in *B*. *cinerea* under GLU and TCW condition, as sole carbon source. (**a**) Biological process classification, and (**b**) Molecular function classification. Relative abundance, on the y-axis, refers to the percentage of membrane-associated phosphoproteins identified in each category relative to the total number of phosphoproteins GO annotations.

To determine the biological relevance of the identified phosphorylated membrane proteins of *B*. *cinerea* that were affected by the changes in the carbon source used, exclusive or over-expressed proteins identified under each condition were categorized according to their specific gene ontology (GO) annotations, by Biological Process (BP) and Molecular Function (MF) (Fig. 4). The GO classification by BP (Fig. 4a) showed 13 different categories present in only one of the carbon sources; one of these (autophagy) appeared only under the GLU condition, and the remaining 12 categories, including cell death, appeared only under the TCW condition. There were other categories that appeared under both conditions, but with several different subcategories under each of the two conditions assayed (Supplementary Data Table S4); among these categories are: (i) small molecule metabolic process; (ii) sulphur compound metabolic process; and (iii) response to stress. Under the GLU conditions the category “small molecule metabolic process” included “acetyl-CoA biosynthetic process from pyruvate”; in contrast, under TCW this category included “sulphur amino acid biosynthetic process”. The category “sulphur compound metabolic process” under the GLU condition presented the subcategory “acetyl-CoA biosynthetic process from pyruvate”; conversely, under TCW this category showed the three subcategories “sulphur amino acid biosynthetic process”, “sulphate assimilation” and “hydrogen sulphide biosynthetic process”. Finally, the category “response to stress“ presented phosphoproteins related to response to oxidative stress only under the TCW condition. The presence of membrane-associated phosphoproteins related to autophagy under the GLU condition agrees with previous reports, which illustrate the importance of the regulation of this catabolic process in the progress of infection in pathogenic fungi^29^. Additionally, under GLU the existence of the subcategory “acetyl-CoA biosynthetic process from pyruvate”, derived from the presence of the phosphoprotein pyruvate dehydrogenase (PDH), suggests that PDH plays a pivotal role in the regulation of the pyruvate metabolism, and therefore in pathogeny, coinciding with previous results reported in *Fusarium graminearum*^30^. Similarly, the presence of phosphorylated proteins identified under the TCW condition, related to “cell death”, “sulphur amino acid biosynthetic process” and “hydrogen sulphide biosynthetic process”, suggests that the regulation of these processes could be essential in the progress of the infection, agreeing with previous reports, in which the association of these processes with infection in phytopathogenic fungi has been shown^28,31–33^. According to its Molecular Function (Fig. 4b), the presence of TCW as a sole carbon source seems to increase the phosphorylation of those membrane proteins associated with the endoplasmic reticulum (ER) misfolded or unfolded protein accumulation response. These proteins are included into 4 Molecular Function categories (Unfolded protein binding, ubiquitin-like protein binding, mRNA binding and peptidase activity) and into 1 category from Biological Process classification (protein binding). These Molecular functions and the Biological Process mentioned are related to the unfolded protein response (UPR), the endoplasmic reticulum-associated degradation pathway (ERAD) and ubiquitin-dependent autophagy elimination of proteins aggregates, which are known to be connected^34–36^. UPR, ERAD and its cooperation are known to be essential for infection processes in several fungi pathogenic to human and plants^37–39^, but need to be elucidated in *B*. *cinerea*.

### Protein interaction analysis

In order to understand better the processes differentially affected by the type of carbon source, we performed a protein interaction analysis using the STRING database and Cytoscape^40,41^. Performing this analysis we searched our identified proteins against *B*. *cinerea* proteins in the STRING database and predicted their physical and functional interactions using seven types of evidence. By using the exclusive and over-expressed proteins in each condition, the analysis returned a network of 108 nodes and 262 connecting edges for TCW (Supplementary Information Fig. S2a) and a network of 14 interacting nodes and 11 connecting edges for GLU (Supplementary Information Fig. S2b). These networks were used to perform a deeper analysis in order to identify highly related interactive proteins (clusters) by using MCODE^42^. Only one cluster was identified for GLU (Table 1), which contains proteins involved in the pyruvate metabolism; this finding coincides with the results from the GO annotation and differential pattern of phosphorylation in common proteins. This result reinforces the importance of the pyruvate metabolism regulation in pathogeny. Under the TCW condition 7 clusters were identified (Table 2); one of these was related to the proteasome, agreeing with the GO results described above, that showed under the TCW condition phosphoproteins related to the UPR or ERAD, which are known to be essential for infection processes in several pathogenic fungi^37,38^.

**Table 1:** Components of the single protein cluster discovered under the GLU condition using MCODE.

STRING	Description	Accession no.	Go/KEGG function*
EDN24165	Pyruvate carboxylase	G2XQQ6	gluconeogenesis, pyruvate metabolic process
EDN33174	Pyruvate dehydrogenase E1 component subunit alpha	G2Y0M8	glycolytic process, acetyl-CoA biosynthetic process from pyruvate/Biosynthesis of secondary metabolites
EDN32877	Similar to acetyl-CoA carboxylase	G2YUC1	acetyl-CoA carboxylase activity, fatty acid biosynthetic process

*The information presented in the column “GO/KEGG function” is obtained from GO and KEGG analysis.

**Table 2:** Components of the 7 protein clusters discovered under the TCW condition using MCODE.

	STRING	Description	Accession no.	GO/KEGG function*
cluster 1	EDN28004	Proteasome subunit alpha type	XP_001553547.1	Proteasome
	EDN24656	Putative uncharacterized protein (579 aa)	G2XPD2	Proteasome
	EDN21254	Putative uncharacterized protein (4066 aa)	XP_001557651.1	Ubiquitin mediated proteolysis
	EDN33567	26S proteasome regulatory subunit RPN2	M7TYC	Ubiquitin mediated proteolysis
	EDN21482	26S protease regulatory subunit 6A	G2YEW	Proteasome regulatory subunit
Cluster 2	END32763	Serine/threonine-protein kinase/ ribonuclease Ire1	G2YTQ5	Protein processing in endoplasmic reticulum/ apoptosis/ kinase/ endoribonuclease activity
	EDN32693	Similar to SNF2-family ATP dependent chromatin remodelling factor snf21	G2YD0	regulation of transcription
	EDN17927	Putative nuclear transcription factor and subunit b-3 protein	M7UH44	regulation of transcription /carbon catabolite activation of transcription
	EDN31104	hypothetical protein BCIN_06g05520	XP_001559090.2	cell growth mode switching, monopolar to bipolar
	EDN26108	Non-specific serine/threonine protein kinase	M7UIQ7	protein kinase
	EDN18852	Mitogen-activated protein kinase hog1 (354 aa)	W8NY90	MAP kinase activity
	EDN19343	Putative serine threonine-protein kinase ppk6 protein	M7V0C1	protein kinase activity
	EDN25239	Bcmyo2	XP_001555378.2	myosin motor involved in actin-based transport of cargos
	EDN17515	BcCRZ1, transcription factor Zn, C2H2	G2XZL7	regulation of transcription/ calcium-mediated signalling
	EDN27443	Ca/CaM-dependent kinase-1 (348 aa)	M7TEN3	calcium calmodulin-dependent protein kinase
Cluster 3	EDN25628	Similar to Grp1p	G2Y921	single-stranded telomeric DNA binding, nuclei acid binding, nucleotide binding
	NIP1	Eukaryotic translation initiation factor 3 subunit	A6S043	Translation
	EDN32817	40S ribosomal protein S12	G2YHW7	Translation
	EDN18720	hypothetical protein BC1G_02869	XP_001558205.1	Translation
	EDN30082	40S ribosomal protein S5 (213 aa)	G2XS65	Translation
	EDN24481	Putative glycine-rich RNA-binding protein	M7TY50	nucleic acid binding, nucleotide binding
Cluster 4	EDN20852	hypothetical protein BC1G_14514	XP_001547112.1	tRNA processing,
	EDN30347	Putative uncharacterized protein	G2Y5Q9	ribosomal subunit export, ribosomal large subunit assembly/translation
	EDN21035	hypothetical protein BC1G_14760	XP_001546946.1	
Cluster 5	EDN20861	Calcium permease	M7TNR0	Sodium, calcium and potassium:proton antiporter activity
	EDN29923	Similar to Na(+)/H(+) antiporter	G2XU17	sodium and potassium ion export across plasma membrane, response to osmotic stress,
	EDN22251	hypothetical protein BCIN_14g04570	XP_001546156.1	Potassium and calcium-transporting ATPase activity
Cluster 6	EDN26839	hypothetical protein BC1G_06858	XP_001554270.1	translation
	EDN18858	glycosyltransferase family 35 protein	G2Y3B5	glycogen catabolic process
	EDN33365	Glycogen synthase/ glycosyltransferase family 3 protein	G2Y3W1	glycogen biosynthetic process
Cluster 7	EDN25463	Putative uncharacterized protein	G2Y9J7	
	EDN31314	Coronin	M7TUZ2	endocytic vesicle, actin cortical patch, regulation of Arp2/3 complex-mediated actin nucleation
	EDN18812	Similar to actin binding protein	G2Y3G4	actin cytoskeleton organization, actin cortical patch assembly
	EDN25070	Similar to serine/threonine protein kinase	G2YC92	protein kinase activity
	EDN26422	hypothetical protein BCIN_13g05360	XP_001554782.2	GTPase activity
	EDN24879	Putative uba ts-n domain containing protein	M7TUH0	actin cortical patch, mating projection tip, cellular bud neck, cellular bud tip

*The information presented in the column “GO/KEGG function” is obtained from GO and KEGG analysis.

### Pathways analysis

With the object of revealing the differences between the two conditions assayed in the biological pathways expressed, a pathway analysis of proteins identified (exclusive and overexpressed) was performed using KEGG^43^. The KO numbers assigned by BlastKOALA 2.1 were mapped to the network datasets KEGG pathway, Reconstruct Pathway and Search&Color Pathway; the latter was used to search against all pathway maps in the “bfu organism (*B*. *cinerea*)” category. The results from using the Reconstruct pathway tool were divided into 2 categories. The category of “Genetic information” (Supplementary Information, Fig. S3) corroborated the presence of phosphorylated membrane proteins involved in functions related to the proteasome, ubiquitin-mediated proteolysis and protein processing in endoplasmic reticulum under the TCW condition, matching the previous GO annotation and MCODE results. The importance of the UPR and ERAD in the pathogeny of *Aspergillus fumigatus*, *Cryptococcus neoformans* and *Alternaria brassicola* has been described previously^37–39^. However, the role in *B*. *cinerea* remains to be elucidated. The category “Environmental information processing” (Supplementary Information, Fig. S4) showed the existence of membrane-associated phosphoproteins related to mTOR and cAMP signalling pathways under the GLU condition. These signalling pathways are known to be implicated in the regulation of autophagy^29^. The presence of phosphorylated membrane proteins related to this biological process under GLU has also been demonstrated in our GO results. Taken together, these results suggest that autophagy is an important route in the regulation of *B*. *cinerea* pathogeny, as previously reported^44,45^. Search&Color Pathway results (Supplementary Data Table S5) showed similar results, supporting: (i) the importance of regulation of the UPR, with the presence under the TCW condition of phosphoproteins implicated in the proteasome, ubiquitin-mediated proteolysis and protein processing in endoplasmic reticulum; and (ii) the importance of the regulation of autophagy, by the presence under the GLU condition of phosphoproteins related to autophagy.

### Proteome Mining

#### Signal transduction

The signalling cascade is essential in the pathogeny because it links the fungal response with the changes in the surrounding environment. Qualitative analyses of proteins identified in each assayed condition suggested possible alterations in five of the most important signalling networks of *B*. *cinerea*, with the strongest evidence of changes in the Small GTPase, MAPK cascade and Ca2+ mediated signal. The total number of signalling phosphoproteins exclusively identified or overexpressed under TCW and GLU was 25 (10%) and 10 (15.6%), respectively. The stress-activated mitogen protein kinase SAK1 (membranome; phosphoproteome; potentially associated to membrane) was identified commonly in both conditions assayed, agreeing with our previous analysis in *B*. *cinerea*^11,12^, but it presented a different phosphorylation pattern depending on the carbon source. Sak1 is a well-known protein of the MAPK cascades that is phosphorylated in response to osmotic and oxidative stress by the MAPKK Bos5^13^. A protein similar to Bos5 (novel; peripheral/cytosolic) was identified only under the TCW condition. Bos5 was not previously identified in our analysis of *Botrytis cinerea*^11,12^, but was reported among the *B*. *cinerea* phosphoproteins by Davanture, M. *et al*.^10^. In addition, the presence of a type 2 C Ser/Thr phosphatase similar to PTC3 (membranome; potentially associated to membrane) under the TCW condition, but not under the GLU condition, may indicate a variation in the MAPK signalling pathway depending on the carbon source used. In relation to the Small GTPase signalling pathway under the TCW condition we found a larger number of phosphorylated membrane proteins related to this signalling network, including the p21-activated protein Cla4 (membranome; phosphoproteome; potentially associated to membrane), and two proteins that could be implicated in the regulation of Rho1: a protein similar to SAC7 (membranome; potentially associated to membrane); and a protein similar to RhoGEF (XP_001561237.2; phosphoproteome; potentially associated to membrane). In yeast, Rho1p is required for regulating the organization of the actin cytoskeleton, the activity of the (1,3)-β-D-glucan synthase and the cell wall integrity^46^. In addition, a different ArfGEF (Guanine nucleotide exchange factor of the ADP ribosylation factor) was identified under both conditions assayed; a protein similar to SYT1 (Bcsyt1) (novel; potentially associated to membrane) was identified under TCW; and a putative guanyl-nucleotide exchange factor protein similar to the yeast SEC. 7(Bcsec. 7) (novel; potentially associated to membrane) was identified under GLU. SEC. 7 is implicated in yeast autophagosome phagophore formation^47^. The last signalling system that presented more identified phosphoproteins under TCW was the Ca2+ mediated signal pathway, which presented, among others, the Calcineurin-responsive zinc finger transcription factor CRZ1 of Botrytis (BcCrz1)^48^ (novel; secreted protein), which is implicated in growth on poor media, hyphal morphology, stress response, cell wall and membrane integrity, and virulence^13^. BcCRZ1, the orthologue of the S. cerevisiae CRZ1 transcription factor, is known to be regulated by phosphorylation/dephosphorylation, being transported to the nucleus to perform its function when is dephosphorylated by calcineurin (CN) phosphatase^48^. Additionally, there were two other phosphoproteins related to the Ca2+/CN-signalling cascade under TCW: (i) BcCMK1 (calcium/calmodulin-dependent protein kinase) (membranome; phosphoproteome; potentially associated to membrane), the orthologue of the yeast CMK1, which is known to be phosphorylated and activated in response to Ca^2+^ counteracting the CN function^49^; (ii) CND8 (novel; potentially associated to membrane), a calcineurin dependent (CND) gene that is upregulated by inhibition of CN^50^. So, the presence of the phosphorylated form of BcCRZ1, BcCMK1 and CND8 indicates a negative regulation feedback of the Ca^2+^/CN-signalling cascade under the TCW condition. It has been described that the inhibition of CN and the deletion of BcCRZ1 resulted in decreased expression levels of botrydial biosynthetic genes^48,51^. Therefore, the negative regulation of BcCRZ1 and CN is consistent with the previously described inhibition of botrydial production under the TCW condition^52^.

All these differences between the signal transduction phosphoproteins identified under each condition suggest that these phosphoproteins play a vital role in the pathogeny through the process that activates them or are activated by them. A set of proteins similar to protein kinase or hypothetical kinases have been identified under TCW condition, which have not been identified in our previous works^11,12^. These proteins could have important function in the adaptation of the fungus to the environmental conditions, constituting an interesting starting point for further studies.

#### Autophagy

Identified phosphorylated membrane proteins involved in autophagy have special relevance because it is known that induction, by nutrient starvation, of macroautophagy (hereafter autophagy) plays an important role in the formation of infection structures (appressorium) in plant pathogenic fungi, and therefore in determining the outcome of the penetration and pathogeny^29^. Under GLU, as a constitutive stage, as sole carbon source and according to the GO and/or KEGG annotation, 8 phosphorylated membrane-associated proteins (exclusive and overexpressed) related to autophagy were identified. Three of them (Atg3, Atg17 and Atg22) were described as ATG proteins, and the remaining five were identified as Avt3/4 (SLC36A), Kcs1, Vps41, ArfGEF and PDK1 (or PDPK1). The Atg3 protein is part of the ubiquitin-like Atg8 conjugation system that allows efficient vesicle expansion during autophagosome formation, and the Cvt pathway^29,53^. In filamentous fungi, Atg17 is specific and required for autophagy induction^54^. In yeast, the Atg17-Atg31-Atg29 complex is present as a stable complex under both vegetative and starvation conditions, but phosphorylations of Atg29 and Atg31 generate an active Atg17-Atg31-Atg29 complex upon starvation conditions in autophagy induction^29,55^. Atg22, Avt3 and Avt4, are vacuole permeases involved in the transport of amino acids produced in the final stage of autophagy^29,56^. Another vacuole protein identified in GLU and related to autophagy is Vps41, which is involved in docking and fusion of the double membrane vesicle with the vacuolar membrane during autophagy and the Cvt pathway^29,57^. Thus the presence of the phosphorylated forms of Atg3, ATG17, Atg22, Avt3/4 and VPS 41 could be due to: (i) the constitutive activity of the Cvt pathway, because Atg3, Atg22, Avt3/4 and VPS 41 are required for both the Cvt pathway and autophagy, and the constitutive presence of the Atg17-Atg31-Atg29 complex; or (ii) the phosphorylation of Atg3, Atg22, Avt3/4 and the autophagy-specific protein Atg 17 could imply the inactivation of autophagy under nutrient-rich conditions (GLU)^29,55,56^. Finally, the other phosphoproteins identified under GLU: KCS1, the SEC. 7 superfamily member ArfGEF, and PDK1, are associated with the regulation of autophagy. KCS1 is an IP6K (inositol-hexakisphosphate 5-kinase) that converts Inositol hexakisphosphate (IP6) to 5-Diphosphoinositol pentakisphosphate (5-IP7), and in yeast has been shown to play a role in macroautophagy^58^. The union of 5-IP7 to AKT1 causes the inhibition of this protein^59^, activating autophagy by the inhibition of mTORC1 in the mTOR signalling pathway. The SEC. 7 superfamily ArfGEF is a GTP-exchange factor that catalyses the activation of the ADP-ribosylation factor family of small GTPases (ARFs). In the yeast *Saccharomyces cere*visiae three proteins containing SEC. 7 domains are required for autophagy by the activation of Arf1 and Arf2 GTPases^47,60^. In mammalian cells, two ArfGEF members of the SEC. 7 superfamily are inhibited by phosphorylation^61^. The last identified phosphoprotein was mammal PDK1-like (phosphoinositide-dependent protein kinase). In some eukaryotes phosphorylated PDK1 is a positive regulator of the TOR pathway, activating mTORC1 and inhibiting autophagy^59,62,63^. These data suggest that, under GLU as sole carbon source, phosphorylated ArfGEF (inactive form) and PDK1 (active form), and possibly also an inactive phosphorylated Kcs1, are inhibiting autophagy in *B*. *cinerea*, indicating a possible role for these proteins in the regulation of autophagy, and therefore in the pathogeny of this fungus. Additionally, the glucose condition is the condition of maximum toxin production^52^, showing that the autophagy route and toxin synthesis seem to be controlled by different routes.

#### Cell death and UPR

The GO and KEGG annotation of our predicted proteins showed 3 proteins related to cell death exclusively identified under TCW conditions. These proteins were predicted to be homologous of *Saccharomyces cerevisiae* proteins Lot6p, Bxi1p and Ire1p, that are known to be related to activation of apoptotic or autophagic cell death in *Saccharomyces cerevisiae*. Ire1p was the only one of these proteins that had not been previously identified in our previous proteomics analysis^11,12^. Phosphorylated Ire1p is the active form of the protein and is related to ER-stress-induced apoptotic cell death in response to an imbalance of unfolded proteins and chaperones (UPR)^64,65^. Lot6p activates apoptosis and reduces the activity of the proteasome in response to oxidative stress^66^. The protein Bxi1p suppresses the activation of apoptotic programmed cell death by the UPR under ER stress and prevents Ire1p phosphorylation^67^. In contrast, overexpression of Bxi1p in plants induces autophagic cell death^68^. The UPR pathway has been reported to be necessary for virulence and during penetration of the plant^37,38^, as well as a possible central regulator of fungal pathogenesis^69^. The presence of these phosphorylated proteins under the TCW condition, which simulates the first contact of the fungus with the host plant, may indicate an activation of cell death in the fungus during penetration; this finding agrees with previous reports that describe the induction of conidia cell death preceding the generation of appressorium during progression of penetration by the fungus^70,71^. In addition, it is known that in eukaryotic cells the balance between autophagy and apoptosis is essential for its survival and for pathogeny^45^. These results highlight the significant role of phosphorylated Lot6p, Bxi1p, Ire1p in the regulation of pathogeny by activation of cell death.

#### Secondary metabolism: PDH

The central carbon metabolism is very important in fungal pathogenesis, so its regulation may be a critical element in the evolution of the infection process. The pyruvate dehydrogenase complex (PDC) converts pyruvate into acetyl-CoA and its regulation is based on the phosphorylation (inactive form) and dephosphorylation (active form) of its pyruvate dehydrogenase (PDH, E1) component. In previous reports it has been shown that, in *Fusarium graminearum*, the deletion of pyruvate dehydrogenase kinase (FgPDK1) resulted in an increase of PDH activity, reduction of the production of sesquiterpene deoxynivalenol (one of most frequently studied mycotoxins in *F*. *graminearum*), and elimination of pathogenicity^30,72^. Those results demonstrate that pyruvate metabolism plays a vital role in the regulation of the growth, toxin production, and pathogenicity in fungi. In our results phosphorylated PDH is overexpressed under the GLU but not under the TCW condition; taken together with our previous report, that shows that the botrydial biosynthesis pathway is inhibited when TCW is present as a sole carbon source, these findings suggest that the production of this toxin is regulated by phosphorylation of PDH^52^. In addition, the presence of the phosphorylated form of BcCRZ1 (inactive) is consistent with the idea that this transcription factor is not the sole regulator of the gene cluster responsible for the biosynthesis of botrydial^48^. Therefore, it is possible that at least two regulation pathways are independently inhibiting the production of botrydial under the TCW condition, which implicate the phosphorylation of BcCRZ1 and PDH.

#### Secondary metabolism: Catalase

It is known that hydrogen peroxide (H_2_O_2_) can affect the tissue of the plant during the infection process, but it also plays an important role in plant defence by inhibiting pathogen infection. So H_2_O_2_ may be destroyed rapidly by plant and fungal catalases (CATs) and peroxidases (POs). Schouten *et al*. (2002) demonstrated the early induction of H_2_O_2_ in tomato tissue infected by *B*. *cinerea* and the elimination of H_2_O_2_ by an extracellular CAT during penetration of the cell wall in the leaf. Subsequent reports have demonstrated that *B*. *cinerea* responds rapidly and efficiently to high levels of H_2_O_2_ added exogenously to the leaves^73,74^. Our results showed a predicted intracellular phosphorylated catalase exclusively identified under the TCW condition, which may indicate an early response to some plant-based elicitor present in TCW. Such a response could represent the initial environmental conditions during penetration of the fungus that prepare the fungal organism for a subsequent increase of H_2_O_2_ levels.

## Conclusion

In this study the phosphomembranome of *B*. *cinerea* has been evaluated in two different pathogenicity states, under the GLU (constitutive stage) and the TCW (virulence inductor) condition. This evaluation revealed a variation in phospho-membranome composition depending on the used carbon source. A total of 1112 proteins have been identified of which 39.21% were not previously identified in the phosphoproteome or the membranome analysis. Among the proteins exclusively identified or overexpressed under each condition, 10.94% and 50.62% under GLU and TCW, respectively, were new proteins not identified in our previous subproteomic analysis. All these new membrane-associated phosphoproteins identified in the present analysis could provide to the research community a new set of targets to the design of novel strategies against *B*. *cinerea*. In addition, these results highlight the usefulness of using the analysis of a combination of two subproteomes to uncover new proteins that were covered up when the subproteome analysis were performed separately. It is interesting to highlight some process which have been showed to be differentially regulated depending on the pathogenicity state in this work: ER stress response (UPR, ERAD, ubiquitin-dependent autophagy elimination of proteins aggregates), cell death; autophagy; pyruvate metabolism (PDH); and response to oxidative stress (catalase). It is known that UPR, ERAD, cell death and autophagy are related to ER stress and their cooperation is essential in the pathogeny/virulence of some fungi, but remains largely unexplored in *Botrytis*. Therefore, the regulation and connection between these biological processes in *B*. *cinerea* must be a key point in the understanding of its infection progress and how to stop it. Finally, a possible regulation of botrydial production by phosphorylation/desphosphorylation of PDH is presented as potential regulation pathway of this toxin, in addition to the regulation performed by CN and BcCRZ1.

## Methods

### Fungal strains and culture conditions

*B*. *cinerea* B05.10 strain (kindly provided by Dr. Paul Tudzunski of the University of Münster, Germany) was used for this study. Conidial stock suspensions were prepared and maintained as previously reported^11^. Two different carbon sources were used: glucose (GLU) (Panreac, Spain) as the constitutive stage; and deproteinized tomato cell walls (TCW) as the virulence inductor, as previously described by Fernandez-Acero *et al*.^75^. In brief, flasks of 500 mL, each containing 250 mL of minimal salt medium (MSM) (50 mM NH4Cl, 7.3 mM KH2PO4, 4.2 mM MgSO4, 6.7 mM KCl, 0.07 mM FeSO_4_) supplemented with 1% of the carbon source assayed, were inoculated with *B*. *cinerea* conidia to a final concentration of 5 × 10^4^ conidia/mL. Four independent replicas were assayed per culture condition. Replicates were incubated in parallel at 180 rpm at 22 °C under alternating 12-h light/dark cycles for 5 days. After 5 days, Phosstop phosphatase inhibitor cocktail (Roche, Basiela, Switzerland) was added to the culture according to the manufacturer’s instructions. Then mycelia were collected by filtration in a 30-µm nylon filter (Sefar Nytal, Switzerland) and stored at -80 °C until use for membrane protein extraction.

### Isolation of phosphorylated membrane proteins

The membrane protein fraction was isolated by the temperature-dependent partition method^76^ with minor modifications^12^. For each biological replica, mycelium was ground into a fine powder with a mortar and pestle using liquid nitrogen. Membrane protein extraction was carried out from 100 mg of powdered mycelium using the “ReadyPrep Protein Extraction Kit (membrane I)” (Bio-Rad, USA) according to the manufacturer’s specifications. A total of five membrane protein extractions were carried out for each biological sample. After protein extraction, only detergent-rich fractions were used. All the replicas from the same biological sample were pooled, precipitated with acetone and quantified using the Qubit 2.0 Fluorometer system (Invitrogen, USA).

Each membrane fraction of 500 μg was dissolved in 6 M urea, 0.2 M NH_4_HCO_3_, then reduced with dithiothreitol (DTT) (10 mM, 37 °C, 60 min), and alkylated with iodoacetamide (20 mM, 25 °C, 30 min). Samples were diluted with up to 2 M urea, digested overnight with Lys-C at 37 °C, and then diluted again 2-fold and digested overnight with trypsin at 37 °C. Peptide mixtures were desalted using a MacroSpin C18 column (The Nest Group, Inc., Southborough, MA). The digested fractions obtained (250 μg) were enriched in phosphopeptides using titanium dioxide (TiO_2_), as previously described^11^. TiO_2_ micro-columns were prepared in gel loading tips (0.5 mg) and were equilibrated with loading buffer (80% ACN in water + 6% TFA). Loading buffer was added to samples and the columns were washed with 80% ACN in water + 0.1% TFA. Finally, phosphopeptides were eluted with 30 μL of elution buffer (5% NH3 in water) into a 1.5-mL tube containing 30 μL of 20% of formic acid in water. Crude phosphopeptide mixtures were desalted using a MacroSpin C18 column (The Nest Group, Inc., Southborough, MA).

### Analysis and identification of proteins by LC‐MS/MS

45% of each enriched phosphopeptide mixture was analysed using a LTQOrbitrap Velos Pro mass spectrometer (Thermo Fisher Scientific, San Jose, CA) coupled to a nano-LC (Proxeon, Odense, Denmark) equipped with a reversed-phase chromatography 25 cm column with an inner diameter of 75 µm, packed with 1.9 µm C18 particles (Nikkyo Technos, Japan). A gradient of 3 to 35% acetonitrile in 360 min with 0.1% formic acid was used, at a flow rate of 250 nL/min. The Orbitrap Velos was operated in positive ion mode with nanospray voltage set at 2.2 kV and source temperature at 325 °C. The instrument was externally calibrated using Ultramark 1621 for the FT mass analyzer. An internal calibration was performed using the background polysiloxane ion signal at m/z 445.120025 as the calibrant. The instrument was operated in data-dependent mode. In all experiments, full MS scans were acquired over a mass range of m/z 350–2000 with detection in the Orbitrap mass analyzer at a resolution setting of 60,000. For each MS scan, the 20 most intense ions with multiple charged ions above a threshold ion count of 5000 were selected for fragmentation using collision-induced dissociation with multistage activation (activation of neutral losses of 98, 65.4, 49, 32.7) at a normalized collision energy of 35% in the LTQ linear ion trap. All data were acquired with Xcalibur v2.2 (Thermo Fisher Scientific, United States). Protein identification was performed with the Proteome Discoverer software suite v.1.4.0.288 (Thermo Fisher Scientific, United States) using MASCOT v2.3 (Matrix Science, United Kingdom) as search engine and a *B*. *cinerea* NCBI database including the most common contaminants (45755 entries; strains B05.10, T4 and BcDW1). Carbamidomethylation for cysteines was set as the fixed modification, and acetylation in protein N-terminal, phosphorylation in Ser, Thr and Tyr, and oxidation of methionine were set as variable modifications. Peptide tolerance was set at 7 ppm in MS, and at 0.5 Da in MS/MS mode. The maximum number of missed cleavages was set at 3. Peptides were filtered based on a false discovery rate (FDR) lower than 1%. Phosphosite location was assigned by the PhosphoRS algorithm. Areas under the curve for the identified phosphopeptide were obtained with the “Precursor Ions Area Detector” algorithm from the Proteome Discoverer software. A fold change value and a Student’s t-test for the phosphopeptides that were in at least three replicates of the four biological replicates in the two conditions was calculated to pinpoint differentially abundant phosphopeptides (p-value < 0.01). Obtained p-values were adjusted for multiple testing with the q-value method of Storey^77^. Only those phosphopeptides with differences in the GLU/TCW ratio (R) of <0.66 and >1.5, with p-value < 0.01, and with q-value < 0.02 were considered as true differentially abundant, and retained for further analyses. For the presence/absence analysis, a protein was considered exclusive to one phenotypical condition if it was present in at least three of the four biological replicates, and was not detected in any replicate of the other condition. No statistical analysis was performed for proteins that exhibited an absence/presence pattern among conditions.

### Bioinformatics analysis

To predict protein topology several different algorithms were used. TMHMM Server v.2.0^78^ was used to predict the transmembrane domain. To detect N-terminal secretory signal peptides, proteins were submitted to the SignalP 4.1 server^79^. Predictions of non-classical protein secretion were performed using predictions for mammalian sequences in the SecretomeP 2.0 server^80^. The Pred-GPI^81^ and big-PI Fungal predictors^82^ were used for the glycosylphosphatidylinositol (GPI) anchored protein prediction. Lastly, to detect prenylated, myristoylated and palmitoylated proteins, the GPS-lipid 1.0 (medium threshold) was used^83^. Additionally, the PrepS-Prediction of Protein Prenylation^84^, NMT-The Myr predictor (fungi specific) and CSS-Palm 4.0 (medium threshold)^85^ were used to predict prenylated, myristoylated and palmitoylated proteins sites, respectively. The STRING protein interaction database version 10.5 (https://string-db.org/)^40^ was used to generate a protein interaction network of the phosphoproteins identified (medium confidence _ 0.4). The protein-protein network obtained was then imported into Cytoscape (version 3.6.1)^41^ and the clustering algorithm MCODE (version 1.5.1) was performed to identify potential functional clusters^42^. We performed a blastp search of the FASTA files containing our identified protein sequences against a database constructed from the complete *Botrytis cinerea* (strain B05.10) ASM83294v1 proteome from the ENSEMBL release 39 (found here: ftp://ftp.ensemblgenomes.org/pub/fungi/release-39/fasta/botrytis_cinerea/pep/). The parameters for the blastp search were: e-value = 1e-3 and max_target_seqs = 1. GO annotations of the proteins selected from the database used were assigned to their related proteins. The GO classification of proteins identified according to their involvement in a biological process (BP) and molecular function (MF) was carried out using the Agbase web server^86^. KEGG annotations of all proteins identified were run with BlastKOALA 2.1^87^. The KEGG annotations obtained were mapped to the network datasets KEGG pathway, using two KEGG mapper v3.1 tools of the KEGG database^43^: Reconstruct Pathway and Search&Color Pathway. The last tool was used to search against all pathway maps in the “bfu organism (*B*. *cinerea*)” category.

## Supplementary information

Supplementary Figures

Supplementary table S1

Supplementary table S2

Supplementary table S3

Supplementary table S4

Supplementary table S5

## Data Availability

The datasets generated during the present study are available in the PRIDE repository, [https://www.ebi.ac.uk/pride/archive/] with the dataset identifier PXD010961.
