# Supplementary material for: Proteomic study of the membrane components of signalling cascades of Botrytis cinerea controlled by phosphorylation
Source: Sci Rep. 2019 Jul 8;9:9860. doi: 10.1038/s41598-019-46270-0 (PMC6614480; doi:10.1038/s41598-019-46270-0)
Supplement: Supplementary file 1 — Supplementary Figures [file 41598_2019_46270_MOESM1_ESM.pdf]

Proteomic study of the membrane components of signalling cascades of *Botrytis cinerea* controlled by phosphorylation

Almudena Escobar-Niño[1+], Eva Liñeiro[1+], Francisco Amil[2], Rafael Carrasco[1], Cristina Chiva[3,4], Carlos Fuentes[5], Barbara Blanco-Ulate[6], Jesús M. Cantoral Fernández[1], Eduard Sabidó[3,4], and Francisco Javier Fernández-Acero[1*]

[1] Andalusian Center for Grape and Grapevine Research (IVAGRO), Microbiology Lab, University of Cadiz, Puerto Real, 11510, Spain
[2] Bioinformatics Unit, SCAI, University of Córdoba, Ramón y Cajal Building, Rabanales Campus, 14071 Córdoba, Spain
[3] Proteomics Unit, Centre for Genomic Regulation (CRG), 08003, Barcelona, Spain
[4] Proteomics Unit, Universitat Pompeu Fabra (UPF), Barcelona, 08003, Spain
[5] Proteomics Unit, SCAI, University of Córdoba, Ramón y Cajal Building, Rabanales Campus, 14071 Córdoba, Spain
[6] Department of Plant Sciences, University of California, Davis, CA 95616, USA
*franciscojavier.fernandez@uca.es
+these authors contributed equally to this work

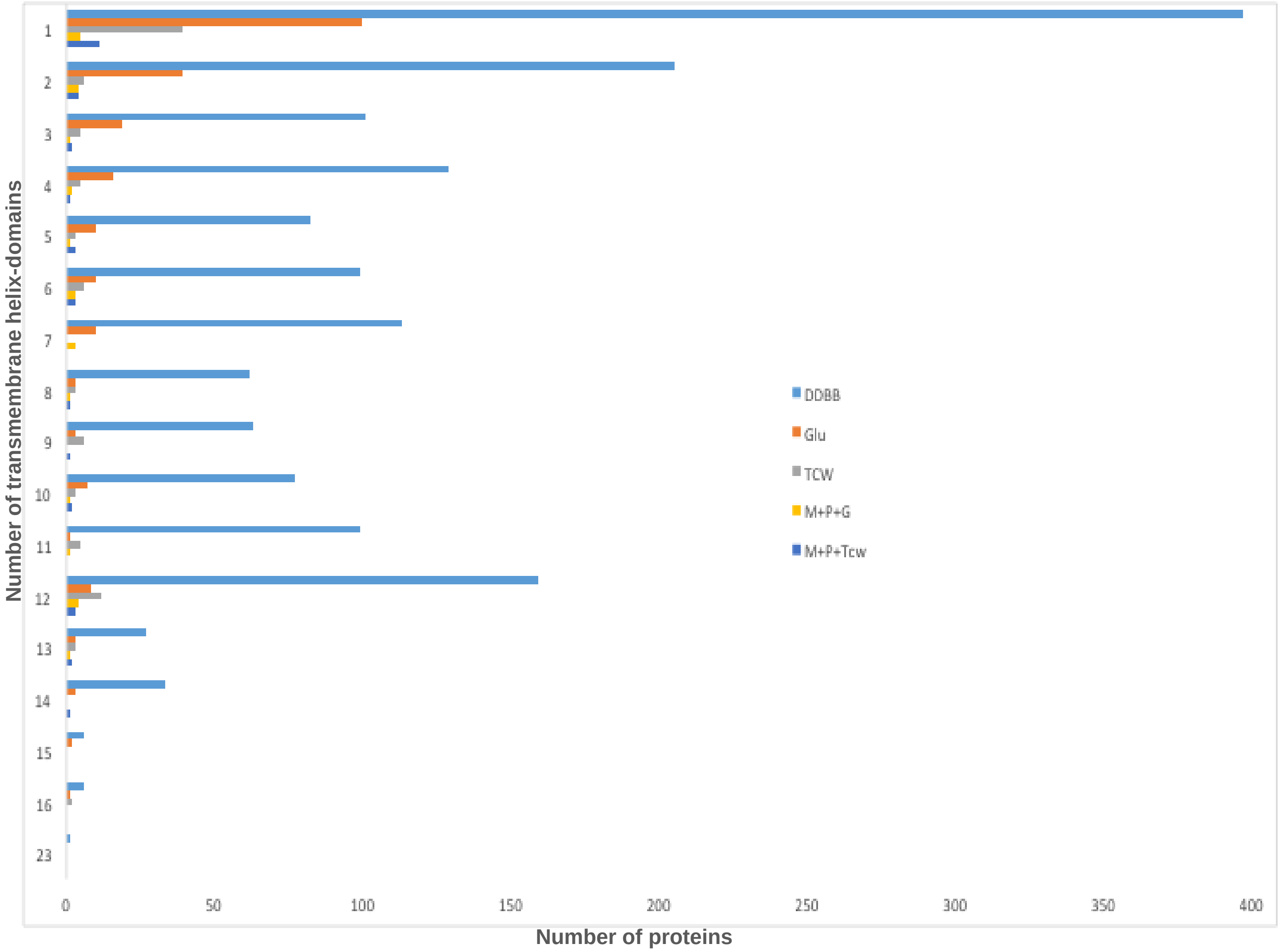

Supplementary Figure S1. **Frequency distribution of the number of transmembrane helix-domains observed in *B. cinerea* .** Comparision of *B.cinera* phosphomembranome under the GLU condition (M+P+G) and the TCW condition (M+P+Tcw) versus number of transmembrane helix-domains predicted for : (DDBB) the *B. cinerea* genome ; (Glu) *B. cinerea* membranome under the GLU conditions; (TCW) *B. cinerea* membranome under the TCW condition.

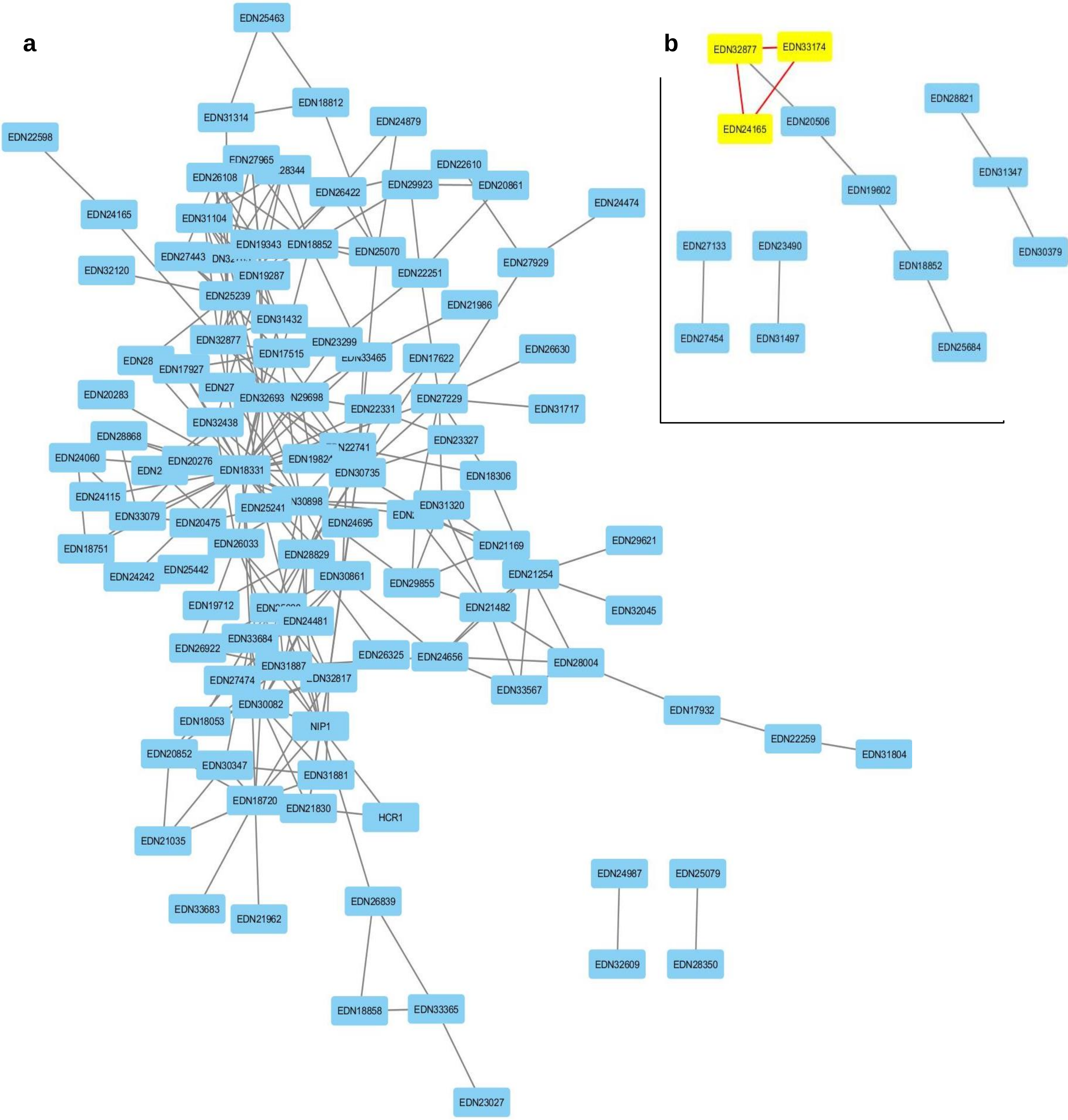

Supplementary Figure S2. Proteins interaction network of proteins exclusively identified and over-expressed under the TCW (a) and the GLU (b) condition, using STRING database and cytoscape. Only proteins that show interactions are displayed. The cluster of proteins with high degree of interaction level according to the MCODE algorithm under the GLU condition is shown in different colour (yellow).

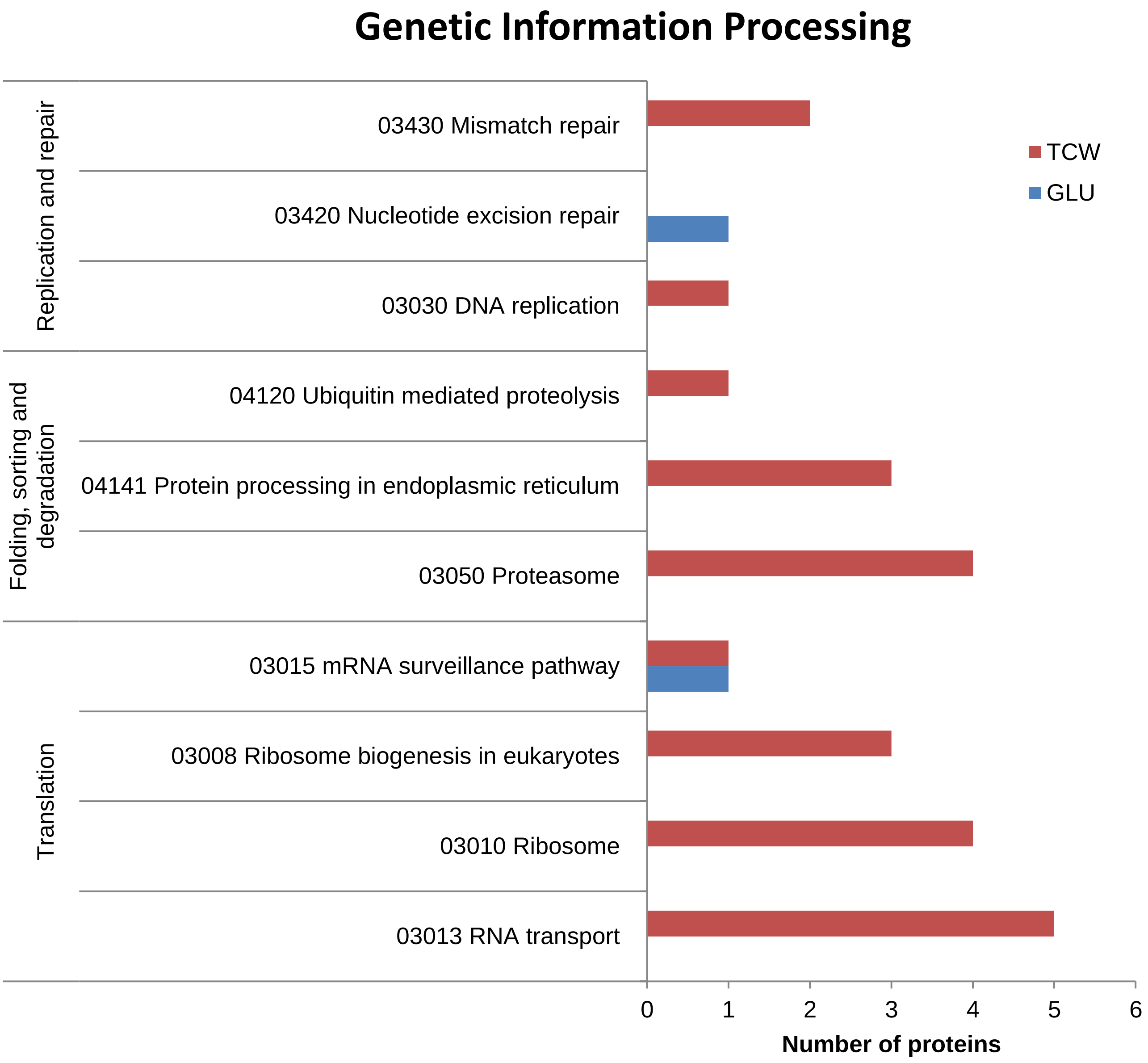

Supplementary Figure S3. Genetic information processing category results using the KEGG Reconstruct pathway tool. Comparison of exclusive or over-expressed proteins under GLU as sole carbon source (blue) and TCW as sole carbon source (red).

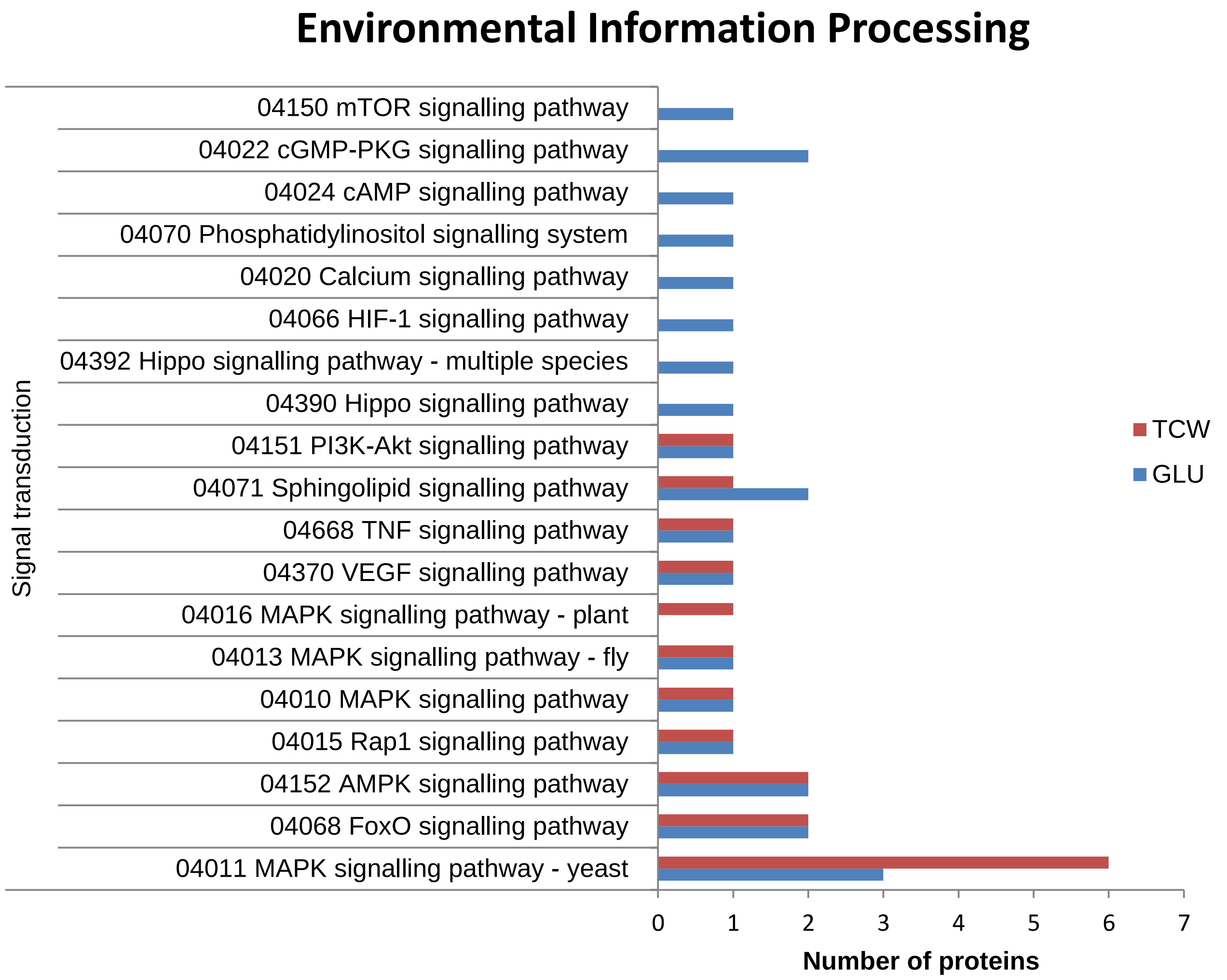

Supplementary Figure S4. Environmental information processing category results using the KEGG Reconstruct pathway tool. Comparision of exclusive or over-expressed proteins under GLU as sole carbon source (blue) and TCW as sole carbon source (red).
